# Skin Diseases and Syphilis

**Published:** 1893-10

**Authors:** 


### *SELECTIONS AND ABSTRACTS.*

### SKIN DISEASES AND SYPHILIS.

EDITED BY DR. M. B. HUTCHINS.

### TREATMENT OF CERTAIN SKIN AFFECTIONS BY THYROID FEEDING.*

Under this title, Dr. Arthur T. Davies, of London, has an article in the September, '93, *British Journal of Dermatology.* Lately there have been reports, especially in German literature, of the beneficial action of extracts of the thyroid gland of some of the domestic animals in myxedema. Dr. Davies was induced to try the treatment in other diseases of the skin. The first case, psoriasis of only three weeks duration, was cured by the use of tabloids containing the extract. One a day was given. "Cure" in eight weeks. No relapse.

The second case, also psoriasis, had lasted three years. Usual treatment beneficial only up to a certain point. One tabloid each day. Practically cured in three months.

The third case was one with an universal xerodermatous condition, with marked ichthyosis of the knees. Under same treatment as the other cases the first condition almost entirely disappeared, and the ichthyotic state was cured.

The fourth case was one of eczema of twelve years' standing, with acute exacerbations. There was also improvement in this case.

Dr. Byrom Bramwell, of Edinburgh, also reported some cases of sporiasis treated with thyroid extract. Was led to try the remedy in psoriasis from having noticed the "exfoliation of skin in myxedema cases treated with thyroids."

* This paper was read in Dermatological Section of British Medical Association, August, 1893.

Case 1 "cured" in three months. Slight recurrence a month later.

Case 2 "cured" in two months.

Case 3 "cured" in two months.

Two out of four other cases benefited.

---

### DISINFECTION OF WOUNDS.

Schimmelbusch spoke on this subject at the twenty-second meeting of the German Surgical Congress (*Deutsche Med. Ztg.*, 1893).

The trial of disinfecting materials had hitherto always been made on "dead" substances, as bouillon, silk threads, etc. Doubtful if results so obtained would apply in living man. The speaker had experimented by making wounds upon animals, putting infective material in them and then applying immediately the disinfectant. The result was the failure to affect the infection in the least. The explanation was that the infection carriers penetrated so quickly that the disinfectants could never more overtake them. Other experiments showed that the germs entered the interstices of the tissues with the greatest imaginable rapidity.—*Monatshefte für Prak. Derm., XVII., No. 4.*

[Another argument in favor of *asepsis* as opposed to *antisepsis*.]

---

### CEREBRAL SYPHILIS.

Cropf (*Münich Med. Wochenschr.*, 1892, No. 11) reports case of a child of thirteen weeks of apparently healthy parents attacked with severe dyspeptic symptoms. "Atrophy, dropsy and convulsions lead to death." Autopsy revealed appearances which could only have been produced by syphilis of the brain. Later it was learned that the father had syphilis eight years before, but had since shown no symptoms.—*M. für Prak. Derm., XVII., No. 4.*

---

### PAGET'S DISEASE OF THE NIPPLE.

Dubois-Havenith (*La Polilicnique,* No. 5, 1893) presented a case of Paget's disease in a patient of eighty years, which began four years before with eczematous appearances. Later a typical carcinoma of the breast developed, with infiltration of corresponding axillary glands and neuralgic pains in the nape of the neck. Psorosperms were demonstrated microscopically. Treatment was total extirpation of the breast. (Does not say what was done with the axillary glands. ED.)—*M. für Prak. Derm., XVII., No. 4.*

Whether the bodies seen in this disease in molluscum epitheliale and epithelioma are psorosperms or other epithelium, or whether they have any causative influence is yet an undecided question.—ED.

---

### TREATMENT OF VASCULAR NÆVI (TELANGIECTASES) WITH BICHLORIDE OF MERCURY AND COLLODION.

Coesfeld (*Der. Arztt. Prakt.*, 1893, No. 13) employs corrosive sublimate 1 part to collodion 8. It is applied with a brush (camel's hair), going a little outside the borders of the lesion. Quickly dried, by blowing, and one or two more layers applied. In ten to twelve days the eschar falls, leaving a dry, white, superficial, smooth scar. Repetition of treatment if necessary. Very large lesions should be treated by rings of the application from without inward. He warns against actual cautery, and fire using nitric acid (why?). Large nævi should be excised.—*M. für Prak. Derm., XVII., No. 4.*

---

### ITEMS FROM NEW YORK DERMATOLOGICAL SOCIETY, 224TH REGULAR MEETING.

*Case of Scrofulous Gummata.*—Dr. Fordyce presented an anemic girl, aged twenty, who came of a tuberculous family. Over the lower extremities she had a number of subcutaneous lesions which, on first view, strongly suggested syphilitic gummata. A few of them had broken down and were discharging curdy pus; others were semi-fluctuating, while a few were quite hard and covered by healthy epidermis. In order to render the diagnosis certain, she had been given the iodide of potassium. Under the use of this drug the lesions became inflamed and the ulceration extended. The iodide was stopped and the lesions were treated locally by iodoform in connection with tonics and cod-liver oil. A marked improvement in her local and general condition was soon apparent.

Dr. Allen concurred in the diagnosis.

Dr. Klotz remarked that the bright red color of the nodes of the skin in the case did not suggest scrofulous gummata, which usually appeared almost pale or of a more bluish color, but looked more like erythema nodosum. The fact, however, that some of the nodes contained pus, and on breaking down had left characteristic ulcers, did not allow of any doubt of the correctness of the diagnosis.

*Case of Ringworm of the Mucous Membrane of the Lip.*—Presented by Dr. Robinson.

The patient, a young man, showed a patch of ringworm extending upon the mucous membrane of the lips as far as the line of contact when the lips were slightly brought together. It was present in both the upper and the lower lip.

Dr. Cutler said he was especially interested in the case, as he had recently treated a young man in private practice for ringworm of the face and neck, where one of the lesions had extended from the lip into the mucous membrane of the mouth. As ringworm affecting mucous surfaces was so rare a disease, even stated by some authors as never occurring, he had intended to report the case.

Dr. Sherwell had never before seen ringworm on the mucous membrane of the lips, but he had seen in sycosis parasitaria the process running up into the mucous membrane of the nostrils, or at least it had so appeared to him clinically.

Dr. Allen spoke of the possibility of molluscum contagiosum occurring upon the vermilion border, as instanced in a case of his own, and saw no reason why trichophytosis might not do the same.

Dr. Robinson said he had presented the case on account of its seat on a mucous membrane, where there did not exist any corneous cells; it seemed, therefore, that here the elements of the trichophyton had lived in cells, the majority of which still had a nucleus and in a part to be regarded as composed of living tissue; hence the trichophyton fungus can live in living tissue, as maintained by himself several years ago.

Dr. Sherwell, in confirmation of his previous remarks, said that he had frequently seen favus develop on spots where there was no epidermis at all.

Dr. Lustgarten had never seen a case of trichophytosis on a mucous membrane.

Dr. Elliott would ask, in connection with this case, whether any one of the members present had observed those cases of ringworm of the palms and soles, which closely resembled syphilitic eruptions. Zambaco had pointed out numerous cases in the Hôpital St. Louis, which had been regarded as syphilitic affections, and had been unsuccessfully treated, but in which he had demonstrated the trichophyton, and they had healed rapidly under antiparasitic treatment with chrysarobin and resorcin. He himself had observed such cases, which did not improve under mixed treatment. He had found the spores and mycelia of the trichophyton in the scales, and the lesions healed speedily under antiparasitic treatment.—*From the N. Y. Journal of Cutaneous and Genito-Urinary Diseases*, Sept., '93.

